# The natural catalytic function of *Cu*GE glucuronoyl esterase in hydrolysis of genuine lignin–carbohydrate complexes from birch

**DOI:** 10.1186/s13068-018-1075-2

**Published:** 2018-03-19

**Authors:** Caroline Mosbech, Jesper Holck, Anne S. Meyer, Jane Wittrup Agger

**Affiliations:** Center for Bioprocess Engineering, Department of Chemical and Biochemical Engineering, Technical University of Denmark, Søltofts Plads 229, 2800 Kgs. Lyngby, Denmark

**Keywords:** Glucuronoyl esterases, CE15, LCC, Glucuronoxylan, Aldouronic acids, Lignin

## Abstract

**Background:**

Glucuronoyl esterases belong to carbohydrate esterase family 15 and catalyze de-esterification. Their natural function is presumed to be cleavage of ester linkages in lignin–carbohydrate complexes particularly those linking lignin and glucuronoyl residues in xylans in hardwood.

**Results:**

Here, we show for the first time a detailed product profile of aldouronic acids released from birchwood lignin by a glucuronoyl esterase from the white-rot fungus *Cerrena unicolor* (*Cu*GE). *Cu*GE releases substrate for GH10 endo-xylanase which results in significantly increased product release compared to the action of endo-xylanase alone. *Cu*GE also releases neutral xylo-oligosaccharides that can be ascribed to the enzymes feruloyl esterase side activity as demonstrated by release of ferulic acid from insoluble wheat arabinoxylan.

**Conclusion:**

The data verify the enzyme’s unique ability to catalyze removal of all glucuronoxylan associated with lignin and we propose that this is a direct result of enzymatic cleavage of the ester bonds connecting glucuronoxylan to lignin via 4-*O*-methyl glucuronoyl-ester linkages. This function appears important for the fungal organism’s ability to effectively utilize all available carbohydrates in lignocellulosic substrates. In bioprocess perspectives, this enzyme is a clear candidate for polishing lignin for residual carbohydrates to achieve pure, native lignin fractions after minimal pretreatment.

**Electronic supplementary material:**

The online version of this article (10.1186/s13068-018-1075-2) contains supplementary material, which is available to authorized users.

## Background

Efficient and complete enzymatic degradation is an essential prerequisite in the utilization of lignocellulosic material for production of energy and value-added biorefinery products. However, cross links in plant cell walls, such as lignin–carbohydrate complexes (LCCs) are obstacles for selective separation and isolation of lignin in enzymatic conversion of plant biomass [1].

Glucuronoxylan present in the secondary cell walls of hardwoods primarily consists of xylan (β-1 → 4-linked d-xylosyl) substituted with α-1 → 2 -linked 4-*O*-methyl-d-glucuronosyl residues [2, 3]. Lignin–carbohydrate ester linkages are formed between aliphatic alcohols in lignin and 4-*O*-methyl-d-glucuronic acid residues of glucuronoxylan [4, 5]. In addition, the bifunctional nature of ferulic acid [6] is assumed to contribute to linkages between the lignin moiety (via ether bonds) and arabinoxylan via esterifications to arabinosyls, hence representing another “LCC” component [7, 8]. It is hypothesized that the 4-*O*-methyl-d-glucuronoyl comprising ester linkages can be enzymatically hydrolyzed in nature by glucuronoyl esterases (GE), a relatively new family of esterases assigned to the CAZymes family 15 under carbohydrate esterase (CE15) (http://www.cazy.org/) [9]. The first CE15 was discovered in the cellulolytic system of the basidiomycete fungus *Schizophyllum commune* in 2006 [10]. At present, the CE15 family contains eight characterized proteins. Of these, the crystal structures of two have been determined [11, 12]. Until now, all glucuronoyl esterases have been characterized using a limited number of synthetic model substrates and very little is known about the biological function of these esterases. Studies on model substrates have revealed a significant importance of the 4-*O*-methyl group on the glucuronoyl residue for catalytic activity [13, 14]. Glucuronoyl esterase from *Schizophyllum commune* has been found to exclusively attack esters of 4-*O*-methyl-glucuronoyls and exhibits no acetyl xylan esterase or feruloyl esterase activity on model substrates [10]. Similar catalytic activity on low and high molecular mass polymeric methyl esters of glucuronoxylan indicates the potential ability of glucuronoyl esterases to act on large substrates, potentially releasing high molecular weight products. This is supported by the active site being exposed on the surface of the protein [15]. However, studies on the biological function of glucuronoyl esterases and any possible correlations between proposed groupings based on bioinformatics and functional differences have been hindered by the lack of natural substrates amendable to hydrolysis by this type of enzyme [16].

High heterogeneity and recalcitrance of lignocellulosic biomass together with low concentration of LCCs have made it difficult to evaluate the effect of glucuronoyl esterases on genuine biomass [1, 14]. The previous two attempts to show GE activity on natural substrates have not demonstrated direct product release. d’Errico et al. treated heat pre-treated corn fiber with complex commercial enzyme preparations Ultraflo^®^ (from *Humicola insolens*) and Cellic^®^ CTec (from *Trichoderma reesei*) supplemented with recombinantly produced glucuronoyl esterases derived from either *Cerrena unicolor* or *T. reesei* and observed a minor increase in the yield of monomeric sugars and glucuronic acid [17]. GE activity has also been detected in several of the commercial complex glucanase preparations from lignocellulose degrading fungi [18] making it complicated to conclude a boosting effect of extra GE supplement. Bååth et al. used two substrates originating from spruce and birch for testing glucuronoyl esterase activity and interpreted a decrease in substrate size by SEC and an increase in carboxylic acid concentration by NMR as evidence for GE activity [19].

We hypothesized that the concentration of LCCs could be increased via biomass fractionation after pretreatment. Enrichment of LCCs in the substrate would make it possible to demonstrate that the glucuronoyl esterase catalyzes the release of products directly from a genuine lignin-rich fraction prepared from birchwood and verify the putative natural action of the enzyme.

## Results

The gene encoding the glucuronoyl esterase from the white-rot fungus *Cerrena unicolor* (*Cu*GE) was successfully expressed in *Pichia pastoris* and purified (see Additional files 1, 2). Glucuronoyl esterase activity was confirmed by monitoring the degradation of benzyl d-glucuronate by LC–MS (see Additional file 3). Furthermore, no endo-xylanase or acetyl xylan esterase activity was observed (see Additional files 4, 5).

A lignin-enriched substrate was prepared from raw birchwood by thermal ethanol extraction (see Additional files 6, 7, 8). The generated lignin-rich precipitate (LRP) served as substrate for *Cu*GE and was hypothesized to contain the natural substrate for glucuronoyl esterase in high concentration (for suggested sketch structure of LRP see Additional file 6). The chemical composition of LRP and raw birchwood was determined by acid hydrolysis (Table 1) and confirmed an enrichment of glucuronoyl substituted xylan and lignin with practically no structural glucan in comparison to the raw birchwood substrate.

**Table 1:** Comparison of relative composition of raw birchwood and lignin-rich precipitate (LRP) after pretreatment

	Structural arabinan	Structural 4-*O*-Me-glucuronoyl	Structural glucan	Structural xylan	Lignin	Structural acetate
mg/g DM						
Raw birchwood	4.49 ± 1.12	n.d.	449.03 ± 26.33	297.08 ± 20.85	198.36 ± 1.97	2.75 ± 0.05
LRP	0.08 ± 0.02	2.39 ± 0.30	0.51 ± 0.07	11.43 ± 2.3	902.05 ± 27.91	0.81 ± 0.17

Values obtained by acid hydrolysis represent relative concentrations as an average of three replicates ± standard deviations based on dry matter of each fraction (mg/g DM). See Additional file 2 for complete description of methods. *n.d.* not determined

**Fig. 1:**
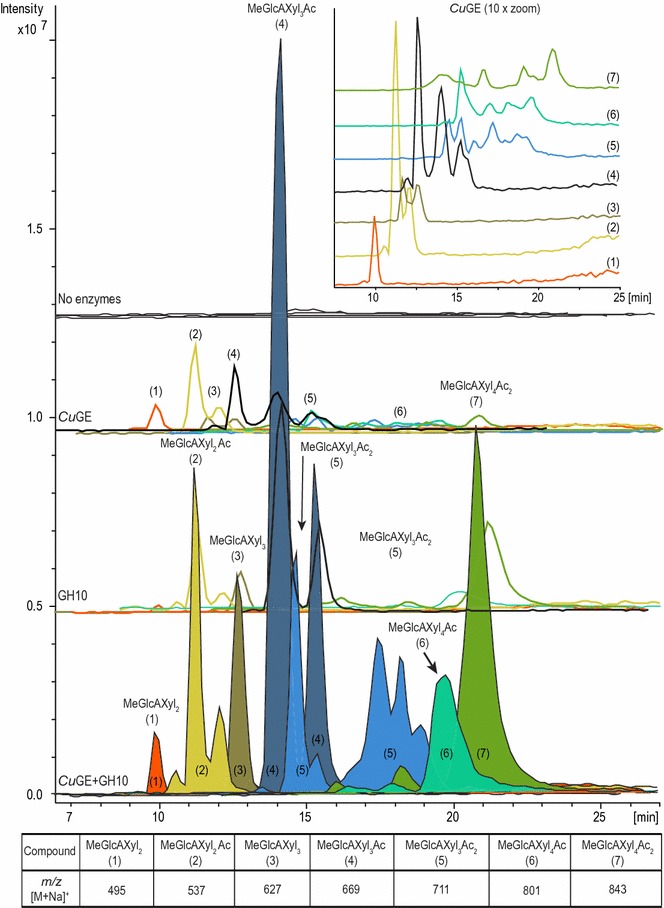
LC–MS chromatograms of enzymatically released sodium adduct of aldouronic acids. Charged product profiles generated from treatment of LRP with either *Cu*GE, GH10 endo-xylanase or a combination of the two (presented on the same intensity scale and displayed with an offset between enzyme combinations). The product profiles show a mixture of acetylated aldouronic acids containing 4-*O*-methyl-glucuronosyl (MeGlcA) ranging from DP 3 to DP 5. Each product is assigned with a number and overall compound composition according to the molecular mass. Several compound masses gave rise to several peaks, i.e., compound (5) indicating structural isomers as a result of differences in substitution pattern. The zoom in the upper right corner shows an enlargement of the product profile resulting from treatment with *Cu*GE alone

*Cu*GE catalyzed release of a mixture of acetylated aldouronic acids upon reaction on LRP (Fig. 1). These results are the first reported example of genuine product release by a glucuronoyl esterase from a complex LCC substrate derived from hardwood. The products released by *Cu*GE strengthen the general hypothesis that glucuronoyl esterases are capable of hydrolyzing ester-linked LCCs of glucuronoxylan and lignin [10]. Also, action by GH10 endo-xylanase resulted in release of acetylated aldouronic acids and neutral xylo-oligosaccharides from LRP (Fig. 1 and Additional file 9). The product release is consistent with partial glucuronoxylan degradation. The addition of *Cu*GE to the GH10 endo-xylanase enzyme reaction significantly increased the total release of the aldouronic acids and verifies that glucuronoxylan in the lignin-rich precipitate was present in both an esterified and a non-esterified form. According to LC–MS analysis, the product profile consisted of products ranging from DP 3 to DP 5 (mass table in Fig. 1) and each product mass gave rise to several peaks indicating various structural isomers of each component (Fig. 1). Even after complete deacetylation with NaOH, the enzyme reaction products released by *Cu*GE resulted in several peaks per mass, probably a result of structural isomers. These isomers might be a result of varying location of the glucuronoyl substitutions and demonstrate that the enzyme most likely attacks the ester bonds randomly, i.e., independently of the glucuronoyl substitutions pattern (see Additional file 10). MS/MS data were consistent with the expected fragmentation patterns of aldouronic acids and xylo-oligosaccharides (see Additional files 11, 12).

**Fig. 2:**
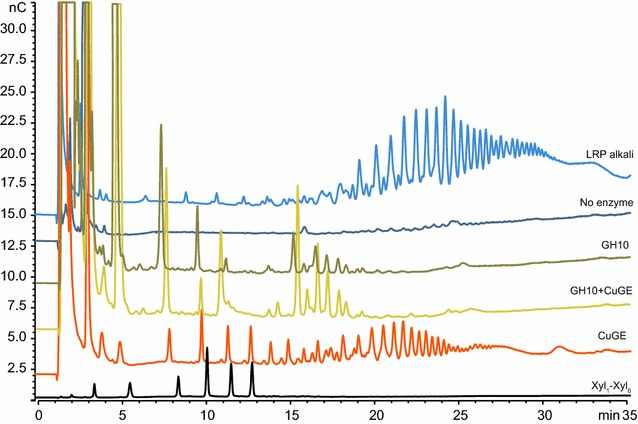
HPAEC-PAD chromatograms of enzyme reaction products and LRP alkali. Comparison of enzyme reaction products released from LRP by *Cu*GE, GH10 endo-xylanase or both from reactions with 5 mg/mL substrate. The chromatograms show a trace of neutral xylo-oligosaccharides from DP1 to DP6 (black) and the NaOH extracted and precipitated glucuronoxylan fraction from LRP as it appears before enzymatic hydrolysis (light blue, LRP alkali, from 37.5 mg/mL substrate). The order of elution on HPAEC is so that neutral components elute first with increasing DP, whereas charged components are retained longer and in this case start to elute after approximately 15 min

The LC–MS is limited in analyzing larger oligomeric enzyme products and to examine the possibility for longer products, the samples were also analyzed by HPAEC-PAD. This analysis confirmed that significantly larger products were also released by *Cu*GE and the products were dominated by aldouronic acids or long neutral products eluting late in the chromatogram (Fig. 2). Comparing the products released by *Cu*GE to the extracted and precipitated glucuronoxylan from LRP and the products released by GH10 alone (Fig. 2, LRP alkali, *Cu*GE and GH10) demonstrates that *Cu*GE is capable of releasing products with a relatively high DP compared to the GH10 endo-xylanase. DP in the original substrate (LRP alkali) appears higher than in the products released by *Cu*GE; however, no endo-xylanase activity has been observed in the initial activity assays of *Cu*GE (see Additional file 5) and the apparently higher DP is a result of higher concentration of aldouronic acids in the LPR alkali fraction. The LRP alkali fraction displays relatively low concentration of short neutral xylo-oligosaccharides due to incomplete precipitation of these species. *Cu*GE’s ability to release long products is in accordance with previous suggestions that glucuronoyl esterases are active on polymeric substrates [15]. GH10 endo-xylanase also released aldouronic acids from LRP (Fig. 2), as affirmed by LC–MS and as expected the products were short compared to the products released by *Cu*GE. *Cu*GE thus appears to catalyze release of products that are in turn substrate for the GH10 endo-xylanase resulting in a significantly enhanced release of short aldouronic acids when the two enzymes act in combination (Fig. 2, *Cu*GE + GH10).

As expected, the GH10 endo-xylanase generated neutral xylo-oligosaccharides (Fig. 2) but it was less expected to observe neutral products released by *Cu*GE and they appear to not originate from any background signal of free xylo-oligosaccharides (Fig. 2, no enzyme). One possible explanation could be that *Cu*GE catalyzes release of neutral arabinoxylo-oligosaccharides after hydrolysis of feruloyl esters in arabinoxylan-like regions of the hemicellulose. According to the chemical composition of LRP, such regions are likely to be present in minor amounts (Table 1) since the compositional analysis of LRP showed minor amounts of arabinose supporting the presence of feruloyl substitutions (see Additional file 7). Previous literature suggests that feruloyl moieties may be incorporated into lignin by ether linkages [8] and if this is the case in the LRP, free feruloyl substituted xylo-oligosaccharides would not be products of GH10 treatment. Assessing the product release catalyzed by *Cu*GE action on wheat arabinoxylan confirmed that the glucuronoyl esterase was capable of releasing ferulic acid in comparable amounts to a genuine CE1 feruloyl esterase (see Additional file 13). However, the *Cu*GE had no activity on methyl ferulate which is in accordance with previous studies on glucuronoyl esterase activity [10]. Because of the surprising feruloyl esterase side activity, it seems plausible that the neutral products released by *Cu*GE could originate from the hydrolysis of feruloyl esters.

**Fig. 3:**
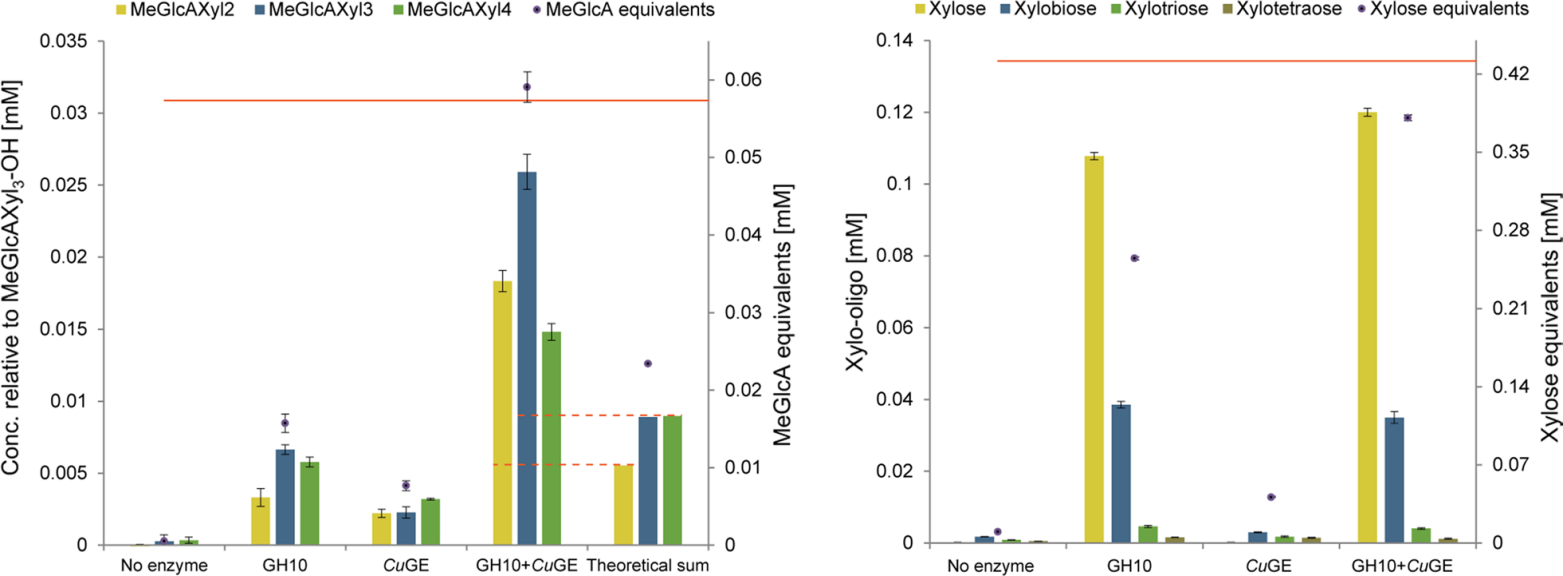
Products released from hydrolysis of LRP by GH10 endo-xylanase and *Cu*GE. **a** Total amounts of aldotriuronic acid (MeGlcAXyl_2_), aldotetrauronic acid (MeGlcAXyl_3_), and aldopentauronic acid (MeGlcAXyl_4_) released by GH10 endo-xylanase and *Cu*GE on LRP quantified relative to reduced aldotetrauronic acid by LC–MS. The theoretical sum of products released by GH10 and *Cu*GE together is calculated as a sum of the products released by the individual enzymes. Dotted lines indicate the level of the theoretical sum on the actual observed release of products. Total amounts of MeGlcA in molar equivalents originating from the aldouronic acids are represented as a scatter with black markers on the secondary axis. The total MeGlcA concentration in the lignin-rich precipitate is illustrated by an orange horizontal line on the secondary axis (for details on quantification see Additional file 2). **b** Total amounts of xylose and major xylo-oligosaccharides (DP2-DP4) released by GH10 endo-xylanase and *Cu*GE on LRP. Xylose concentration quantified by HPAEC-PAD and xylo-oligos (DP2-DP4) quantified on LC–MS. Black scatter markers referring to the secondary axis represent the calculated total release in xylose mole equivalents originating from aldouronic acids, xylose, and xylo-oligos. Total amount of xylose in LRP after acid hydrolysis is indicated by an orange horizontal line. All quantifications are performed in triplicate and depicted with standard deviations. Calculated MeGlcA and xylose equivalents are depicted with pooled standard deviations

Addition of *Cu*GE to the endo-xylanase reaction on LRP resulted in a significant increase in the release of aldouronic acids in comparison to the theoretical sum of charged products released by GH10 endo-xylanase and *Cu*GE individually (Fig. 3a). The synergistic effect was not directly apparent from quantification of the neutral oligosaccharides in the enzyme hydrolysate (Fig. 3b) but this is because a significant portion of xylosyls was present in the aldouronic acids. The effect becomes evident when the total amount of xylose mole equivalents is summarized from both the neutral and charged products (Fig. 3b, black markers on secondary axis). Hence, the synergistic effect appears because *Cu*GE released only glucuronidated oligomeric products that acted as new substrate for the GH10 endo-xylanase. In total, the amount of 4-*O*-methyl-glucuronoyl and xylosyls released by *Cu*GE and GH10 endo-xylanase estimated by mole equivalents from the oligomeric products correspond well to the total amounts present in the starting LRP (Fig. 3, orange horizontal lines, secondary axes). The MeGlcA equivalents released by co-incubation of *Cu*GE and GH10 corresponds to approximately 100% found in the starting material (see Additional file 2 for method of determination). In comparison, GH10 alone resulted in release of only 27% of the MeGlcA equivalents found in LRP. Around 88% of the xylose equivalents found in LRP was released in the reaction with both GH10 endo-xylanase and *Cu*GE in the form of neutral xylo-oligomers and aldouronic acids. 88% xylose release indicates complete removal of glucuronoxylan from LRP when considering the standard deviation of the total xylose determination (see Additional file 7).

## Discussion

In this study, we have positively verified product release catalyzed by glucuronoyl esterase CE15 from *Cerrena unicolor* most likely as a result of cleavage of ester-linked LCCs in hardwood. The extraction method generated a substrate with relatively low background carbohydrate signal by the removal of all cellulose and most of the hemicellulose. Hence, the common recalcitrance parameters in lignocellulose were removed, enabling the detection of enzyme reaction products generated directly by glucuronoyl esterase activity.

The fact that *Cu*GE can release aldouronic acids from the substrate strongly indicates the presence of ester-linked LCCs in the substrate, and is also supported by release of aldouronic acids after direct saponification. GH10 endo-xylanase alone can release aldouronic acids from LRP; hence, not all glucuronoyls are associated with lignin. The glucuronoxylan in LRP appears with a relatively high degree of polymerization as evaluated by HPAEC-PAD (Fig. 2) indicating that the substrate after ethanol extraction is a better representation of the original biomass compared to the synthetic substrates currently used for assessing GE activity. The glucuronoxylan is also highly acetylated as would be expected in the native glucuronoxylan. The degree of acetylation does not seem to affect the activity of *Cu*GE as numerous sizes of aldouronic acids are released (Fig. 2) and various acetylated products and isomers are detected by LC–MS (Fig. 1). The influence of the lignin moiety of the ester linkage on *Cu*GE’s activity is not evaluated here but the lignin structure in LRP is expected to be optimal for *Cu*GE activity because the substrate closely resembles the naturally occurring structure. In previous studies, the ester linkages have been synthesized by esterification to simpler alcohols [20] with little resemblance to lignin. We observe a synergistic activity between *Cu*GE and GH10 endo-xylanase because *Cu*GE releases oligomeric products that serve as new substrate for GH10 endo-xylanase. The synergistic effect is directly observed in Fig. 3a for charged aldouronic acid products where the sum of released MeGlcA equivalents is significantly higher than an additive effect of the GE and GH10 individually. The fact that no apparent synergistic effect on release of neutral oligosaccharides is observed indicates that each xylo-oligomeric chain is linked by several closely positioned glucuronoyl ester linkages creating a heavily glucuronidated substrate for the endo-xylanase. According to the increase in short aldouronic acids, the glucuronoyl substitutions are likely to occur for every 2nd–4th xylosyl residue in certain block regions of the substrate. No oligomeric products carrying two MeGlcA substitutions were detected in the samples incubated with either *Cu*GE alone or GH10 and *Cu*GE together; such specimens might occur in *Cu*GE-hydrolyzed samples but would have too high DP to be directly detected in this setup (Fig. 1). In samples with co-incubation of *Cu*GE and GH10, the products would predominantly be mono-glucuronidated as a result of the endo-xylanase activity. If the distance between glucuronoyl esters in LRP was similar to the average glucuronoyl substitution on glucuronoxylan (1 out of 10 xylosyl residues), the synergistic effect would also be observed directly in the release of neutral xylo-oligosaccharides. Our results prove that the combined action of *Cu*GE and a GH10 endo-xylanase is sufficient to obtain complete release of ester-linked glucuronoxylan from lignin.

It appears that the activity of *Cu*GE is not restricted by steric obstacles such as acetylations compared to GH10 endo-xylanase [21, 22] and this could be related to a surface exposed active site [11] and might also explain why *Cu*GE has feruloyl esterase activity on polymeric substrate. In relation to the natural function of the enzyme, it seems highly relevant that the enzyme possesses side activity towards other ester-linked LCCs in minor amounts that link carbohydrates to lignin [8] (i.e., feruloyl esters) instead of evolving a highly specific esterase for this purpose only. To the best of our knowledge, it has not been investigated if any of the known feruloyl esterases could have activity towards glucuronoyl esters. Altogether our findings contribute to the explanation of the biological function of glucuronoyl esterases as enzymes evolved to enable full utilization of the last remaining residues of carbohydrates in lignocellulosic biomass after removal of the main part of polysaccharides. The biological function of GEs corroborates the presence of several putative GE sequences identified by recent bioinformatics studies in genomes of saprophytic fungi that are highly specialized at growing on the most recalcitrant lignin-containing types of biomass [23, 16].

In an application aspect, glucuronoyl esterases can serve as a tool for enzymatic polishing of lignin in biomass residues where pretreatment has not destroyed ester-linked LCCs. Many pretreatment strategies are focusing on disrupting cellulose crystallinity by employing exposure to high temperatures and alkaline or acidic conditions. Such strategies will cause hydrolysis of most ester-linked LCCs and thereby eliminate the need for glucuronoyl esterases [24]. However, such approaches also cause lignin to condense and thereby decrease the final value and applicability of the lignin product and inhibits the cellulolytic conversion [25]. A pretreatment strategy that is based on the initial selective separation of a relatively pure, native lignin fraction from the major polysaccharides could potentially increase both the value and the applications of lignin and lower the costs of converting cellulose and hemicellulose [26, 27]. Glucuronoyl esterases are highly important enzymes for industrial applications that aim for selective lignin recovery in order to obtain a final high-quality lignin product from hardwood.

## Conclusion

Glucuronoyl esterase *Cu*GE from *Cerrena unicolor* releases aldouronic acids from a lignin-enriched fraction from birchwood most likely as a result of hydrolysis of ester linkages between lignin and glucuronoxylan. The enzyme also releases minor amounts of neutral xylo-oligosaccharides from hydrolysis of feruloyl ester linkages to arabinoxylan-like regions in the substrate. *Cu*GE boosts the activity of GH10 endo-xylanase synergistically by releasing glucuronidated xylo-oligosaccharides that act as new substrate for the GH10 endo-xylanase. In this way, the two enzymes are capable of releasing all neutral and charged xylo-oligosaccharides associated with the lignin-rich birchwood fraction. Further insight into the kinetics and cooperative mechanism of the two enzymes will elucidate the exact biocatalytic degradation and should be pursued.

The action of *Cu*GE demonstrates a biological function of the enzyme as part of the fungal enzyme battery necessary to retrieve all available carbohydrates from the most recalcitrant parts of plant cell walls. The fact that the enzyme exhibits feruloyl esterase side activity signifies that even linkages present in negligible amounts are of importance to the survival of the host organism. Glucuronoyl esterases are clear candidates for polishing of lignin from hardwood and we suggest an approach in pretreatment of hardwood where lignin is extracted prior to hydrothermal pretreatment. In this way, the purity and hence the value and applicability of lignin will increase.

## Methods

### Preparation of LRP

Lignin-Rich Precipitate (LRP) from Norwegian birchwood was prepared as previously reported [28]. 15 g raw birchwood was mixed with 135 mL 50 v/v-% ethanol in a batch reactor (300 mL HC EZE-Seal, Parker Autoclave Engineers, Pennsylvania USA). The reactor was heated to 180 °C and kept at 180 °C for 1 h with stirring at 600 rpm. After extraction, the reactor was cooled and the material retrieved. The pre-treated liquor was separated from the solid biomass by filtration using Ashless 40 filter paper, 8 µm (Whatman). The solids (named Cellulose-Rich Precipitate) were washed with 50 v/v-% EtOH and freeze dried. The pre-treated liquor (280 mL in total including washings) was diluted with three volumes of water (840 mL) resulting in the precipitation of a Lignin-Rich Precipitate (LRP). pH in the pre-treated liquor was measured to 3.8. The suspension of lignin-rich precipitate and the pre-treated liquor was separated by centrifugation. The separated liquor phase consisted of a hemicellulose-rich liquid (HRL). The lignin-rich precipitate was washed in water and freeze dried prior to use for hydrolysis experiments. A schematic overview of the extraction process can be found in Additional file 6. All fractions were subjected to acid hydrolysis for determination of monomeric components (see Additional file 2). A small portion of the LRP was subjected to NaOH treatment in order to release all esterified carbohydrates from the lignin matrix. 0.5 M NaOH was added to approx. 11.25 mg of LRP in suspension in water to reach pH > 11 and kept at room temperature overnight. Hereafter, absolute ethanol was added to a final concentration of 90 v/v-% and kept overnight at 4 °C. The precipitate was retrieved by centrifugation, re-dissolved in water, and analyzed by HPAEC-PAD as LRP alkali and represented the majority of the carbohydrate moiety of the lignin-rich fraction. It is expected that the precipitation of short, linear, neutral xylo-oligosaccharides under these conditions is incomplete. The resulting concentration in the LRP alkali fraction analyzed by HPAEC-PAD originated from 37.5 mg/mL. As described below, the enzyme hydrolysis samples were performed with 5 mg/mL substrate and hence the final concentration of analytes in LRP alkali was approx. 7.5 times higher than in the enzyme hydrolysis samples.

### Enzymatic hydrolysis

Lignin-rich precipitate was suspended in 25 mM sodium acetate buffer pH 6 to a concentration of 5 mg/ml. *Cu*GE (for expression and purification protocol see Additional file 2) and GH10 endo-β1,4-xylanase Shearzyme^®^ 500 L (batch CDN00486) (donated by Novozymes A/S) were added either individually or together to the reactions to a final concentration of 30 mg enzyme protein/g dry matter and 10 mg enzyme protein/g dry matter, respectively, and incubated for 24 h at 50 °C. After reaction, the solid substrate was removed by centrifugation and the supernatant used for analysis.

### HPAEC-PAD

Enzyme reaction products and substrate (LRP alkali) were analyzed by HPAEC-PAD using a CarboPac PA100 (4.6 × 250 mm) and guard (4.6 × 50 mm) on a Dionex ICS3000 system (Thermo Fischer Scientific, Sunnyvale, CA, USA). The column was operated at 1 mL/min with eluent A (water), eluent B (500 mM NaOH), and eluent C (500 mM sodium acetate) according to the following gradient: 0–2 min isocratic 40% B and 1% C, 2–35 min linear gradient to 40% B and 45% C, hereafter immediately shifted to 5% B and 90% C and these conditions were kept for 4 min and ended by shifting back to starting conditions and reconditioning for 6 min.

### LC–MS analysis

Identification and quantification of enzyme reaction products were performed by LC–MS analysis on a UHPLC Dionex UltiMate 3000 system (Thermo Fischer Scientific, Sunnyvale CA, USA) connected to an ESI-iontrap (model AmaZon SL from Bruker Daltonics, Bremen, Germany) operated in MRM mode (multiple reaction monitoring) or fullscan mode. The UHPLC was equipped with a porous graphitized carbon column (Hypercarb PGC, 150 × 2.1 mm; 3 µm, Thermo Fischer Scientific, Waltham, MA, USA) including a guard column of same brand (10 × 2.1 mm). The column was operated at 0.4 mL/min at 70 °C with eluent A (0.1% formic acid) and B (acetonitrile) according to the following gradient: 0–1 min 0% B, from 1 to 15 min linear gradient to 50% B, from 15 to 20 min linear gradient to 80% B, 20–30 isocratic 80% B, hereafter immediately back to starting conditions followed by reconditioning for 10 min.

The ESI was operated in positive mode with spray nebulizer at 3 bar nitrogen, a dry gas flow, and temperature of 12 L/min and 280 °C, respectively. The capillary cap voltage was set to 4.5 kV and end-plate offset of 0.5 kV. Target mass was set to 500 and for MRM conditions, a pre-determined list of ions representing the sodium adducts of exact masses of neutral and charged species was applied: Xyl_2_ (m/z 305), Xyl_2_Ac (m/z 347), Xyl_2_Ac_2_ (m/z 389), Xyl_3_ (m/z 437), Xyl_3_Ac (m/z 479), Xyl_3_Ac_2_ (m/z 521), Xyl_3_Ac_3_ (m/z 563), XylMeGlcA (m/z 231), Xyl_2_MeGlcA (m/z 495), Xyl_2_MeGlcAAc (m/z 537), Xyl_3_MeGlcA (m/z 627), Xyl_3_MeGlcAAc (m/z 669), Xyl_3_MeGlcAAc_2_ (m/z 711), Xyl_4_MeGlcA (*m/z* 759), Xyl_4_MeGlcAAc (m/z 801), and Xyl_4_MeGlcAAc_2_ (m/z 843). CID fragmentation was performed using SmartFrag enhanced amplitude ramping of 100% and helium as the colliding gas. Data analysis and quantification were performed by Compass DataAnalysis 4.2 and Compass QuantAnalysis 2.2 from Bruker Daltonics.

Quantification of enzyme reaction products was done against external calibration curves of authenticated standards of xylobiose, xylotriose, xylotetraose, and reduced aldotetrauronic acid all of which were purchased from Megazyme, Ireland. Samples for quantification purposes were treated with NaOH prior to analysis (and after enzyme reaction) in order to raise pH > 11 and hereby remove all acetylations. This procedure resulted in a much simplified mixture of reaction products mainly consisting of xylose, xylobiose, xylotriose, xylotetraose, aldotriuronic acid, aldotetrauronic acid, and aldopentauronic acid (see Additional file 14). The aldouronic acids were quantified relative to the signal response for reduced aldotetrauronic acid. Elaborate description of the quantification procedure is provided in the Additional files 2, 15.

## Additional files

**Additional file 1.** SDS gel of purified *Cu*GE.

**Additional file 2.** Additional methods.

**Additional file 3.** Assessment of glucuronoyl esterase activity by *Cu*GE.

**Additional file 4.** Assessment of acetyl xylan esterase activity by *Cu*GE.

**Additional file 5.** Assessment of endo-xylanase activity by *Cu*GE.

**Additional file 6.** Schematic overview of ethanol extraction procedure and schematized LRP structure.

**Additional file 7.** Relative composition of four biomass fractions of birchwood.

**Additional file 8.** Mass balance for four biomass fractions of birchwood.

**Additional file 9.** Neutral xylo-oligosaccharides released by *Cu*GE and GH10 endo-xylanase after treatment of LRP.

**Additional file 10.** Chromatogram of *Cu*GE hydrolysed LRP after saponification.

**Additional file 11.** MS/MS of charged products released by by *Cu*GE and GH10 endo-xylanase after treatment of LRP.

**Additional file 12.** MS/MS of neutral products released by by *Cu*GE and GH10 endo-xylanase after treatment of LRP.

**Additional file 13.** Release of ferulic acid by *Cu*GE from water insoluble wheat arabinoxylan.

**Additional file 14.** Complete list of MS precursors and MS/MS fragmentation ions for MRM.

**Additional file 15.** Complete list of adducts used for quantification of enzyme reaction products.
