# Supplementary material for: The natural catalytic function of CuGE glucuronoyl esterase in hydrolysis of genuine lignin–carbohydrate complexes from birch
Source: Biotechnol Biofuels. 2018 Mar 19;11:71. doi: 10.1186/s13068-018-1075-2 (PMC5858132; doi:10.1186/s13068-018-1075-2)
Supplement: Supplementary file 1 — Additional file 1. SDS gel of purified CuGE. [file 13068_2018_1075_MOESM1_ESM.docx]

Additional file 1


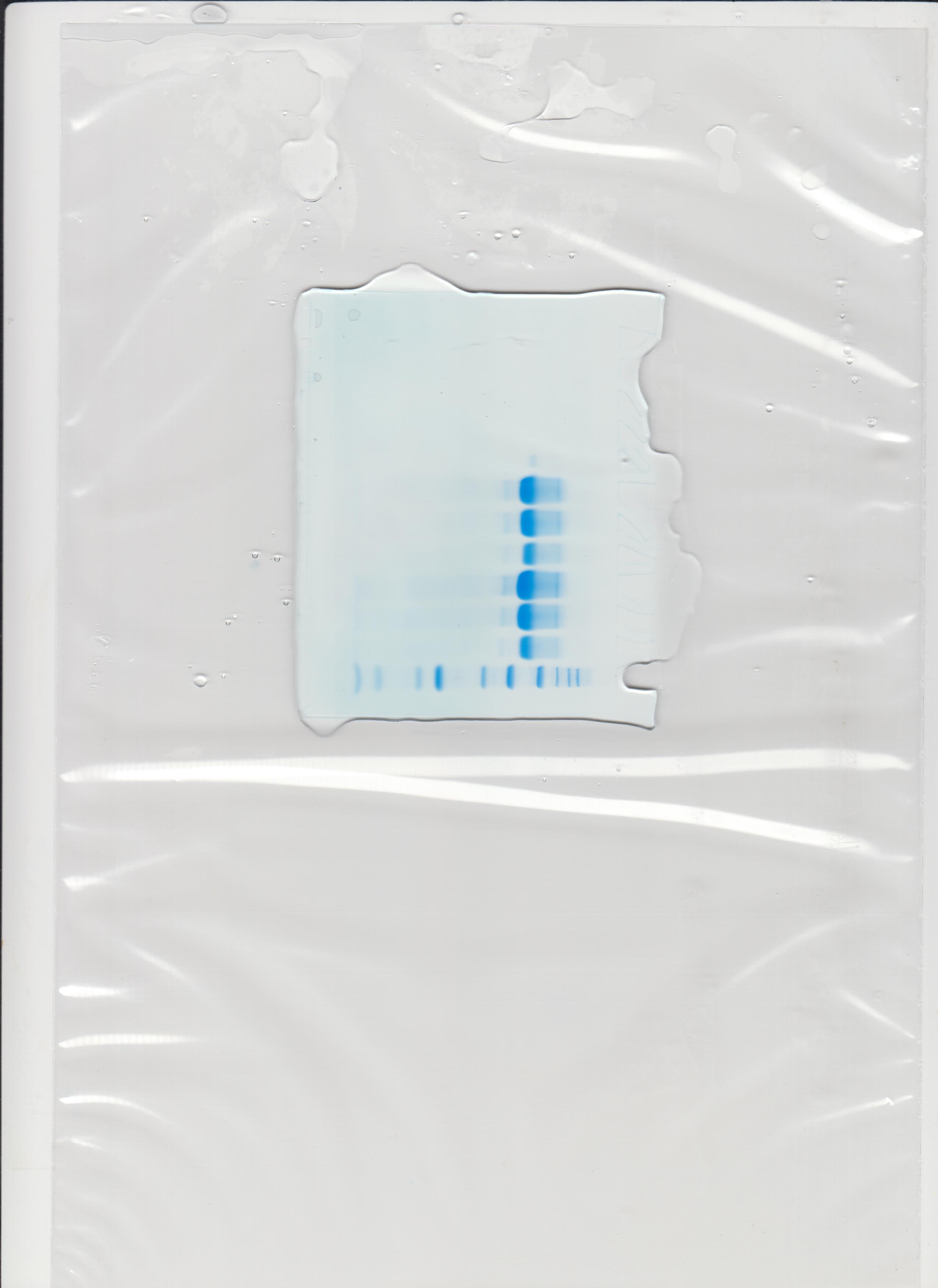


75 kDa

50 kDa

25 kDa

10 µL

5 µL

15 µL

1

2

3

4

Coomassie stained SDS gel of purified *Cu*GE (lane 1-3) in three different concentrations showing a major band at the expected molecular weight of approx. 60 kDa including glycosylations and his-tag (theoretical weight of the protein with no glycosylation is predicted to be approx. 51 kDa). Molecular size ladder in lane 4.
