# Supplementary material for: The natural catalytic function of CuGE glucuronoyl esterase in hydrolysis of genuine lignin–carbohydrate complexes from birch
Source: Biotechnol Biofuels. 2018 Mar 19;11:71. doi: 10.1186/s13068-018-1075-2 (PMC5858132; doi:10.1186/s13068-018-1075-2)
Supplement: Supplementary file 2 — Additional file 2. Additional methods. [file 13068_2018_1075_MOESM2_ESM.docx]

# Additional file 2

# Additional methods

## *Pichia Expression*

Pichia-optimized gene sequence of *Cerrena unicolor* glucuronoyl esterase (*Cu*GE)

Genbank accession number: AIY68500.1 (protein sequence)

GAATTCCAAGCATCCGCTCCTCAATGGGGTCAATGTGGTGGTATCGGTTGGACTGGTCCT
ACTGCCTGCCCTAGTGGTTGGGCCTGTCAGCAATTGAACGCTTACTATTCTCAATGTTTG
CAAGGTGCTGCTCCTGCTCCAGCTAGAACTACTGCTGCTCCACCTCCACCTCCAGCTACT
ACTGCTGCTCCTCCACCTCCAACTACTTCTGCTCCTACTGGTTCTTCTCCAGTTGCTGGT
GCTTGTGGTGCTATTGCTTCTACTGTTCCAAACTATAACAATGCTAAGTTGCCTGATCCA
TTCACTTTCGCTAATGGTACTGCTTTGAGAACTAAAGCTGATTGGTCTTGTAGAAGAGCT
GAAATTTCTGCTTTGATTCAAAACTACGAGGCTGGTACTTTGCCTCCAAAGCCTCCAGTT
GTTACTGCTTCTTTTTCTAAATCTGGTAACACTGGTACTTTGGCTATTACTGCTGGTTTG
TCTAATTCTCAAACTATTAAATTCTCTCCTACTATCTCTTACCCATCTGGTACTCCTCCA
GCTAATGGTTGGCCATTGATTATTGCTTATGAAGGTGGTTCTATTCCTATTCCAGCTGGT
GTTGCTACTTTGACTTACTCTAACTCTGATATGGCTCAACAAAATTCTGCTTCTTCCAGA
GGTCAAGGTTTGTTTTACCAATTGTATGGTTCTACTCATTCTGCTTCTGCTATGACTGCT
TGGGTTTGGGGTGTTTCCAGAATTATTGATGCTTTGGAGATGACTCCTACTGCTCAAATT
AATACTCAAAGAATCGGTGTTACTGGTTGTTCCAGAGATGGTAAAGGTGCTTTGATGGCT
GGTGCTTTCGAAGAGAGAATTGCTTTGACTATTCCACAAGAATCTGGTTCTGGTGGAGAT
GCTTGTTGGAGATTGTCTAAGTACGAGATCGATAACGGTAACCAAGTTCAAGATGCTGTT
GAAATTGTTGGAGAGAACGTTTGGTTCTCTACTAACTTCAACAACTACGTTCAAAAGTTG
CCTACTGTTCCAGAAGATCATCACTTGTTGGCTGCTATGGTTGCTCCTAGAGCTATGATC
TCTTTCGAGAACACTGATTATTTGTGGTTGTCTCCAATGTCTTCTTTCGGTTGTATGACT
GCTGCTCATACTGTTTGGCAAGGTTTGGGTATTGCTGATTCTCACGGTTTTGCTCAAGTT
GGTGGTCATGCTCACTGTGCTTGGCCTTCTTCTTTGACTCCACAATTGAACGCTTTTATT
AACAGATTCTTGTTGGATCAATCTGCTACTACTAATGTTTTCACTACTAACAATCAATTC
GGAAAGGTTCAGTGGAATGCCGCAAATTGGATTACTTGGACTACCCCTACTCTTACTGGT
CTAGA

CE15 glucuronoyl esterase from *Cerrena unicolor* (*Cu*GE) was expressed in *Pichia pastoris* X-33. *Cu*GE has already been characterized by d’Errico et al. in 2015 [1]. Constructs containing the gene encoding *Cu*GE in frame with a C-terminal sequence coding for His-tag were prepared by Genscript, codon optimized for *P. pastoris* and delivered in *p*PICZalphaA vector. Chemically competent *Escherichia coli* DH5α were prepared using Mix & Go E. coli Transformation Kit (Zymo Research, Irvine USA) and transformed with the plasmid DNA. Clones containing the heterologous plasmid were selected on low salt LB-medium containing 25 µg/mL Zeocin as a selective marker. Recombinant plasmid was purified with QIAprep Spin Miniprep Kit (Qiagen, Germany) following manufactures instructions. The plasmid was linearized with SacI and transformed into *P. pastoris* by electroporation according to the manual of EasySelect Pichia expression kit (Invitrogen). Positive clones were selected on YPD plates containing 100 µg/mL Zeocin. Protein expression was verified in small scale fermentation in BMGY and BMMY media with methanol induction according to the user manual form Invitrogen and afterwards performed by 5 L fermentation according to Silva et al 2011 [2]. In short, a 5L Sartorius Biostat Aplus fermenter with basal salt medium was inoculated with an overnight culture of the transformed *Pichia*-strain. Process conditions like pH, agitation speed, oxygen supply, temperature and feeding rates were controlled during the entire fermentation. The total fermentation time was approx. 96 hours and the fermenter was operated in three modes; glycerol batch, glycerol fed-batch and lastly methanol fed-batch for induction. The total glycerol growth phase was approx. 26 hours and was followed by a gradual increase in methanol feed for approx. 16 hours reaching a methanol concentration of approx. 0.5%. The total methanol induction phase lasted for approx. 70 hours. The final working volume of the fermenter was 4 L. When the fermentation was completed, the cells were separated by centrifugation and the fermentation broth was sterile filtered and concentrated by ultrafiltration on a 10 kDa cut-off membrane to a final volume of approx. 80 mL.

## *Enzyme Purification*

*Cu*GE was purified by affinity chromatography on an IMAC-column (HisTrap HP 5 mL column, GE Healthcare) using an Äkta Purifier 100 (GE Healthcare, Uppsala Sweden). Fermentation broth was diluted 5 times in binding buffer (20 mM Na_3_PO_4_, 0.5 M NaCl, 10 mM imidazole pH 7.5), filtered and applied to the column operated at 3 mL/min. Protein was eluted with a gradient from 0 to 100% elution buffer (20 mM Na_3_PO_4_, 0.5 M NaCl, 0.5 M imidazole pH 7.5) over 12 min in 1 mL fractions. Protein purity was assessed by SDS gel (additional file 1). For storage, the buffer was exchanged three times on a 10 kDa spin filter to 20 mM ammonium acetate pH 7, 50 mM NaCl and 10% glycerol. The protein concentration was measured by Bradford assay.

## *Enzyme Activity Assays for CuGE.*

Glucuronoyl esterase activity was assessed using the model substrate benzyl D-glucuronate (Carbosynth, Berkshire UK). Activity was examined on LC-MS with a reverse phase Hypersil GOLD Phenyl column (2.1 x 150 mm; 1.9 µm) (Thermo Scientific) with multiple reaction monitoring (MRM) of *m/z* 307, [M+Na]^+^. The column temperature was 40 °C and flow at 0.4 ml/min running isocratic with 20% acetonitrile and 80% 0.01% formic acid solution. Benzyl D-glucuronate was dissolved in 25 mM sodium acetate buffer to a concentration of 0.05 mg/mL and enzyme added to a final concentration of 2 mg enzyme protein/mg substrate. Reactions were placed in the UHPLCs autosampler at 40 °C and sampled every 6.5 min by direct injection to the column. Enzyme activity was determined as decrease in substrate concentration and external standards were included for quantification. Quantification was performed using Compass QuantAnalysis according to the description in additional file 2 section 5

Acetyl xylan esterase activity was tested using p-Nitrophenyl acetate (Sigma Aldrich, USA) (pNpA) solubilized in DMSO to a concentration of 50 mg/ml. The pNpA solution was diluted 100 times in 25 mM sodium acetate buffer pH 6 and the assay was performed in 96 microtiter plate with each well containing 200 µL substrate solutions and 10 µL enzyme in appropriate dilution to a final enzyme concentration of 10 mg EP/g pNpA. The plate was incubated at 40 °C and absorbance at 405 nm measured every 30 seconds for a total of 20 min. For positive control, a known acetyl xylan esterase; Flavolaschia sp. CE1[3] was used. The CE1 was donated by Novozymes A/S.

Endo-ß-1,4-Xylanase activity was assessed using AZO-xylan from birchwood (Megazymes, Ireland) following the manufactures instructions. A 1% AZO-xylan solution was prepared on a hot-plate stirrer to dissolve the polysaccharide. The assay was performed in Eppendorf tubes containing 400 µL 1% AZO-xylan and 550 µl 50 mM acetate buffer pH 5 at 40 °C 1000 rpm. Reactions were initiated by adding 50 µL enzyme solution in appropriate dilution to a final concentration of 10 mg enzyme protein/g substrate. 50 µL samples were taken every minute for 13 minutes added to 200 µL precipitation solution (100% EtOH) and mixed well. Samples were centrifuges and supernatant aliquoted to a 96-well plate and measured spectrophotometrically at 590 nm. For positive control, GH10 endo-xylanase Shearzyme® 500L was used.

Feruloyl esterase activity was assessed by using methyl-ferulate (CAS no 2309-07-1 from Alfa Aesar) and water insoluble wheat arabinoxylan (P-WAXYI) (Megazyme, Ireland). Methyl-ferulate was dissolved in 50% ethanol at 20 mg/mL and diluted 20 times in 25 mM sodium acetate pH 6 for the activity assay. Insoluble wheat arabinoxylan was suspended to 5 mg/mL in 25 mM sodium acetate pH 6. Both substrates were incubated with *Cu*GE at 30 mg enzyme protein/g substrate at 50 °C for 24 hours. For positive control ferulic acid esterase *An*FAE from *Aspergillus nidulans* (AN.5267.2) [4] was used. Ferulic acid was measured and quantified by LC-MS with a reverse phase Hypersil GOLD Phenyl column (2.1 x 150 mm; 1.9 µm) (Thermo Scientific) with MRM of *m/z* 192.7, [M-H]^-^. For more details on quantification see description in additional file 2 section 5.

## *Sulfuric acid hydrolysis*

Four biomass fractions; the raw biomass before ethanol extraction (raw birchwood), the cellulose-rich precipitate (CRP), the lignin rich precipitate (LRP) and the hemicellulose-rich liquid (HRL) were subjected to acid hydrolysis to determine the composition and content of specific monosaccharides, acetate and Klason lignin. The sulfuric acid hydrolysis was performed according to [5] with small modifications to accommodate small sample amounts and differences in cellulose content. Specifically, approx. 100 mg of dry material of the solid fractions were weighed out in triplicate. Only raw birchwood and CRP samples were subjected to 72% acid, as these were the only fractions containing significant amounts of cellulose. 1 mL 72% H_2_SO_4_ was added to raw birchwood and CRP containing tubes and incubated for 60 min at 30 °C with light shaking. Hereafter 28 mL of water was added to each tube. At the same time triplicate tubes containing either 10 mL 10 mg/mL suspended LRP fractions, 10 mL HRL or 10 mL sugar recovery standard (containing arabinose, glucose, xylose and glucuronic acid; 0.5 mg/mL each) was prepared and 348 µL 72% H_2_SO_4_ added to each tube. All reactions were incubated for 120 min at 121 °C, and hereafter the remaining procedure for determining Klason lignin was identical to [5]. The longer incubation time at 121 °C was done to compensate for the presence of oligosaccharides in samples after incubation for only 60 min. Monosaccharide content was analysed by HPAEC-PAD on a Dionex ICS3000 system (Thermo Fischer Scientific, Sunnyvale, CA, USA) equipped with a CarboPac PA1 column (4.6 x 250 mm) and guard (4.6 x 50 mm) running 1 mL/min at 50 mM NaOH isocratically for 20 min. Xylose concentrations measured in enzyme reaction samples were performed on the HPAEC-PAD as described here. Acetate was determined on a BioRad Aminex HPX87H column (7.8 x 300 mm) operated at 63 °C and a flow of 0.6 mL/min isocratically at 4 mM H_2_SO_4_ on a Shimadzu Prominence HPLC. Detection by refractive index.

4-O-methyl glucuronoyl content was estimated in the lignin rich precipitate (LRP) and the hemicellulose-rich liquid (HRL) according to the following procedure. 5 mg LRP and 5 mL HRL (of 0.35% DM) was saponified in 0.5 M NaOH over night at room temperature in triplicate. Sufficient amounts of NaOH was added to maintain pH>11 and so 25 µL NaOH was added to LRP (1 mL total volume) and 175 µL NaOH was added to HRL (5 mL total volume). Hereafter, 9 mL and 45 mL of absolute ethanol was added to LRP and HRL respectively and left at 4 °C over night to precipitate. The pellet was retrieved after centrifugation and redissolved in 1 mL 25 mM sodium acetate buffer pH 6. 6 µL GH10 endoxylanase in was added in sufficient amount and incubated over night at 50 °C. Enzyme generated aldouronic acids, primarily aldotri- and aldotetrauronic acid in each set of samples were quantified relative to a reduced aldotetrauronic acid by LC-MS. See details below in section 5.

## *Quantification of enzyme reaction products by LC-MS with external calibration*

Quantification of enzyme reaction products was done by LC-MS with external calibration standards. Signal response by mass spectrometry is dependent on ion suppression and ionization efficiency. In order to obtain reliable quantification by mass spectrometry, conditions for calibration and sample should be as comparable as possible. Ionization efficiency is determined by the chemical structure of the compounds and when applying identical or near-identical authenticated standards of the compounds in question the ionization becomes as comparable as possible. In the work here, neutral xylo-oligosaccharides were determined against calibration with authenticated xylobiose, xylotriose and xylotetraose standards. In addition, benzyl D-glucuronate and ferulic acid containing samples were also quantified against calibration with the identical compound in question. The aldouronic acids; aldotri-, aldotetra- and aldopentauronic acid were all quantified relative to the response of reduced aldotetrauronic acid (Megazymes). The relative quantification towards this near-identical compound was necessary due to lack of chemically identical authenticated standards and is expected to give rise to minor deviations concerning the ionization efficiency between the standards and the analytes.

Comparable conditions for ion suppression for standards and analyte samples are achieved by diluting the samples to the extent possible without compromising the lower detection limit. Because the analyte samples consist of a much more complex matrix than the simplified standards it is not possible to completely alleviate ion suppression on the samples, but applying chromatographic separation prior to detection improves the conditions for ion suppression considerably and adds to the effects of dilution. The composition and especially the ion strength of the mobile phase also influence the ion suppression considerably. In the quantifications performed here the standards elute at the exact same time or close to the analytes, hence minimizing any mobile phase effects. In addition, the ionic strength of the mobile phase (0.1% formic acid and acetonitrile) is generally low and adds only little to the total ion statistics.

Enzyme reaction products from LRP and samples for determination of total MeGlcA equivalents have been quantified based on samples acquired in full scan mode because complete quantification is dependent on adduct formation. Accordingly, quantification was done based on the sum of extracted ion chromatograms for each adduct (see additional file 15 for complete list of adducts). The enzyme reaction samples were treated with NaOH prior to analysis for quantification and this decreased the product profile complexity considerably.

Quantification was done using QuantAnalysis software with the peak detection algorithm Version 2.1. S/N ratio at 1, Area threshold and Intensity threshold at 0.1. The generated method included extracted ion chromatograms for each product containing the masses in additional file 15. All peaks were manually inspected and aligned. Aldouronic acids; aldotri-, aldotetra- and aldopentauronic acids were all quantified relative to a linear standard curve for the reduced aldotetrauronic acid. Neutral xylo-oligosaccharides (DP2-DP4) were quantified individually against their respective quadratic standard curves. All quantifications were conducted in triplicate.

1. d’Errico C, Jørgensen JO, Krogh KBRM, Spodsberg N, Madsen R, Monrad RN. Enzymatic degradation of lignin-carbohydrate complexes (LCCs): Model studies using a fungal glucuronoyl esterase from Cerrena unicolor. Biotechnol Bioeng. 2015;112:914–22.

2. Silva IR, Larsen DM, Meyer AS, Mikkelsen JD. Identification, expression, and characterization of a novel bacterial RGI Lyase enzyme for the production of bio-functional fibers. Enzyme Microb Technol. 2011;49:160–6.

3. Agger J, Viksø-Nielsen A, Meyer AS. Enzymatic Xylose Release from Pretreated Corn Bran Arabinoxylan: Differential Effects of Deacetylation and Deferuloylation on Insoluble and Soluble Substrate Fractions. J Agric Food Chem. 2010;58:6141–8.

4. Bauer S, Vasu P, Persson S, Mort AJ, Somerville CR. Development and application of a suite of polysaccharide-degrading enzymes for analyzing plant cell walls. Proc Natl Acad Sci U S A. 2006;103:11417–22.

5. Sluiter A, Hames B, Ruiz RO, Scarlata C, Sluiter J, Templeton D, et al. Determination of Structural Carbohydrates and Lignin in Biomass. Biomass Anal Technol Team Lab Anal Proced. 2004;2011 July:1–14.
