# Supplementary material for: The natural catalytic function of CuGE glucuronoyl esterase in hydrolysis of genuine lignin–carbohydrate complexes from birch
Source: Biotechnol Biofuels. 2018 Mar 19;11:71. doi: 10.1186/s13068-018-1075-2 (PMC5858132; doi:10.1186/s13068-018-1075-2)
Supplement: Supplementary file 3 — Additional file 3. Assessment of glucuronoyl esterase activity by CuGE. [file 13068_2018_1075_MOESM3_ESM.docx]

Additional file 3

Assessment of glucuronoyl esterase activity by *Cu*GE. The enzyme was incubated with benzyl D-glucuronate and the decrease in substrate concentration was followed by mass spectrometry over time (see additional methods file 2 for more details). *Cu*GE is clearly capable of hydrolyzing benzyl D-glucuronate whereas GH10 endo-xylanase cannot hydrolyse the ester. The data are shown as an average of triplicate determinations with ± standard deviations.
