# Supplementary material for: The natural catalytic function of CuGE glucuronoyl esterase in hydrolysis of genuine lignin–carbohydrate complexes from birch
Source: Biotechnol Biofuels. 2018 Mar 19;11:71. doi: 10.1186/s13068-018-1075-2 (PMC5858132; doi:10.1186/s13068-018-1075-2)
Supplement: Supplementary file 4 — Additional file 4. Assessment of acetyl xylan esterase activity by CuGE. [file 13068_2018_1075_MOESM4_ESM.docx]

Additional file 4

Assessment of acetyl xylan esterase activity by *Cu*GE. The enzyme was incubated with pNp-acetate and the release of pNp was measured spectrophotometrically by following the absorbance at 450 nm over time (see additional methods file 2 for more details). *Cu*GE did not release any pNp from the substrate and neither did the GH10 endo-xylanase. A CE1 acetyl xylan esterase (AXE) was included as positive control.
