# Supplementary material for: The natural catalytic function of CuGE glucuronoyl esterase in hydrolysis of genuine lignin–carbohydrate complexes from birch
Source: Biotechnol Biofuels. 2018 Mar 19;11:71. doi: 10.1186/s13068-018-1075-2 (PMC5858132; doi:10.1186/s13068-018-1075-2)
Supplement: Supplementary file 5 — Additional file 5. Assessment of endo-xylanase activity by CuGE. [file 13068_2018_1075_MOESM5_ESM.docx]

Additional file 5

Assessment of endo-xylanase activity by *Cu*GE. The enzyme was incubated with AZO-xylan and the release of blue AZO-dye was measured spectrophotometrically by following the absorbance at 590 nm over time (see additional methods file 2 for more details). *Cu*GE did not release any AZO-dye from the substrate. GH10 endo-xylanase was included as a positive control.
