# Supplementary material for: The natural catalytic function of CuGE glucuronoyl esterase in hydrolysis of genuine lignin–carbohydrate complexes from birch
Source: Biotechnol Biofuels. 2018 Mar 19;11:71. doi: 10.1186/s13068-018-1075-2 (PMC5858132; doi:10.1186/s13068-018-1075-2)
Supplement: Supplementary file 6 — Additional file 6. Schematic overview of ethanol extraction procedure and schematized LRP structure. [file 13068_2018_1075_MOESM6_ESM.docx]

Additional file 6

Schematic overview of ethanol extraction procedure for the generation of a lignin rich precipitate (LRP). Raw birchwood in 50% ethanol was pre-treated in a batch reactor at 180 °C for 1 hour. LRP was obtained by diluting the pre-treated liquor with water resulting in precipitation.


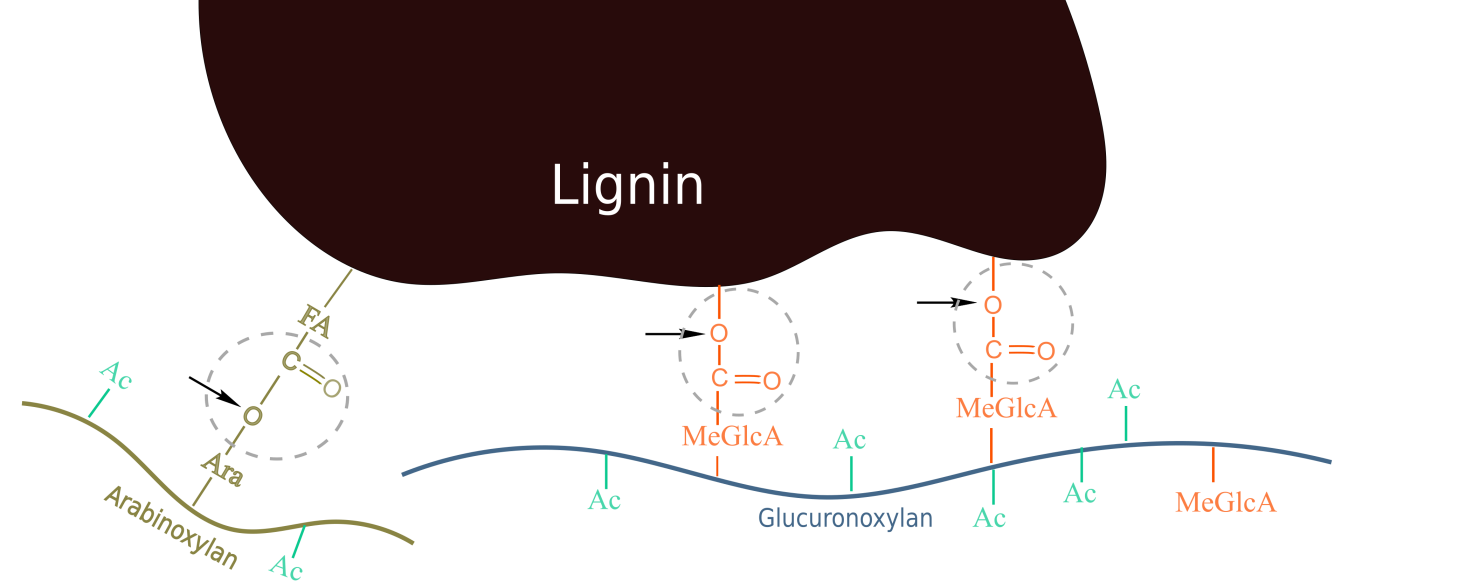


Schematic drawing of suggested structure of LRP. The drawing shows glucuronoxylan chains linked to lignin via ester linkages formed by 4-*O*-Me-glucurnoyl substitutions and arabinoxylan chains linked to lignin via ester linkages formed by feruloyl substutions on arabinofuranosyls. *Cu*GE is expected to target the ester linkages between xylans and lignin as indicated by arrows and cycles on the drawing.
