# Supplementary material for: The natural catalytic function of CuGE glucuronoyl esterase in hydrolysis of genuine lignin–carbohydrate complexes from birch
Source: Biotechnol Biofuels. 2018 Mar 19;11:71. doi: 10.1186/s13068-018-1075-2 (PMC5858132; doi:10.1186/s13068-018-1075-2)
Supplement: Supplementary file 7 — Additional file 7. Relative composition of four biomass fractions of birchwood. [file 13068_2018_1075_MOESM7_ESM.docx]

# Additional file 7

# Relative composition of four biomass fractions of birchwood, before and after ethanol extraction. Results are obtained mainly from sulfuric acid pretreatment and are given as relative concentrations based on dry matter (mg/g DM) as an average of three replicates with standard deviations. The numbers for 4-O-methyl-glucuronoyls are estimated after NaOH treatment, precipitation, enzymatic digestion and relative quantification by LC-MS (see additional file 2). 4-O-methyl-glucuronoyls are only determined for the hemicellulose rich fraction (HRL) and the lignin rich fraction (LRP). Degree of substitution by glucuronoyls on the structural xylan is 0.13 % and 25.1 % for HRL and LRP respectively. n.d.; not determined.

|  | **Structural arabinan** | **Structural**  **4-*O*-methyl-glucuronoyl** | **Structural glucan** | **Structural xylan** | **Lignin** | **Structural acetate** |
| --- | --- | --- | --- | --- | --- | --- |
|  | **mg/g DM** | | | | | |
| Hemicellulose rich liquid (HRL) | 9.60 ±1.92 | 0.9 ±0.02 | 77.81 ±12.06 | 504.96 ±80.66 | 91.69 ±24.81 | 4.90 ±0.20 |
|  |  |  |  |  |  |  |
|  |  |  |  |  |  |  |
| Lignin rich precipitate (LRP) | 0.08 ±0.02 | 2.39 ±0.30 | 0.51 ±0.07 | 11.43 ±2.3 | 902.05 ±27.91 | 0.81 ±0.17 |
|  |  |  |  |  |  |  |
|  |  |  |  |  |  |  |
| Cellulose rich precipitate (CRP) | 0.00 ±0.00 | n.d. | 535.01 ±20.64 | 221.70 ±7.59 | 254.74 ±9.14 | 1.70 ±0.03 |
|  |  |  |  |  |  |  |
|  |  |  |  |  |  |  |
| Raw birchwood | 4.49 ±1.12 | n.d. | 449.03 ±26.33 | 297.08 ±20.85 | 198.36 ±1.97 | 2.75 ±0.05 |
