# Supplementary material for: The natural catalytic function of CuGE glucuronoyl esterase in hydrolysis of genuine lignin–carbohydrate complexes from birch
Source: Biotechnol Biofuels. 2018 Mar 19;11:71. doi: 10.1186/s13068-018-1075-2 (PMC5858132; doi:10.1186/s13068-018-1075-2)
Supplement: Supplementary file 8 — Additional file 8. Mass balance for four biomass fractions of birchwood. [file 13068_2018_1075_MOESM8_ESM.docx]

# Additional file 8

# Mass balance for four biomass fractions of birchwood before and after ethanol extraction. All numbers are given as absolute numbers with two digits and are based on the relative composition given in additional file 7. Each column is summarized for the three pretreated fractions (CRP, HRL and LRP) and related to the starting material (raw) in the balance row. Each row is summarized to the right and balanced against the total amount of material in each fraction. The data inform about the efficiency of the sulfuric acid hydrolysis by which these numbers have been derived and about potential losses during the ethanol extraction process. Relative concentrations in the low range appear as zeros in the table, as they contribute a minority to the entire mass balance (e.i. structural 4-O-methyl-glucuronoyl). In total, the majority of mass can be accounted for in the three different pretreated fractions (CRP, HRL and LRP) except for HRL where approx. 30% material is not found in the analysis. The mass difference may be explained by the presence of xylose degradation products formed during the ethanol extraction[9]. Such degradation products are not quantified in this study.

|  | **Dry matter** | | **Structural arabinan** | **Structural glucan** | **Structural xylan** | **Structural 4-*O*-methyl-glucuronoyl** | **Structural acetate** | **Structural lignin** | **Sum** | **Balance (%)** |
| --- | --- | --- | --- | --- | --- | --- | --- | --- | --- | --- |
|  | | **(g)** | | | | | | | |  |
| **Raw** | | 14.40 | 0.06 | 6.47 | 4.28 | n.d. | 0.04 | 2.86 | 13.64 | 94.72 |
|  | |  |  |  |  |  |  |  |  |  |
| **CRP** | | 8.96 | 0.00 | 4.79 | 1.99 | n.d. | 0.02 | 2.28 | 9.08 | 101.32 |
| **LRP** | | 1.29 | 0.00 | 0.00 | 0.01 | 0.00 | 0.00 | 1.16 | 1.17 | 91.02 |
| **HRL** | | 3.66 | 0.04 | 0.29 | 1.85 | 0.00 | 0.02 | 0.34 | 2.49 | 67.94 |
| **Sum** | | 13.91 | 0.04 | 5.08 | 3.85 | 0.00 | 0.03 | 3.78 | 12.74 | 91.57 |
| **Balance (%)** | | 96.61 | 109.12 | 78.54 | 89.88 | - | 86.51 | 132.39 |  |  |
