# Supplementary material for: The natural catalytic function of CuGE glucuronoyl esterase in hydrolysis of genuine lignin–carbohydrate complexes from birch
Source: Biotechnol Biofuels. 2018 Mar 19;11:71. doi: 10.1186/s13068-018-1075-2 (PMC5858132; doi:10.1186/s13068-018-1075-2)
Supplement: Supplementary file 9 — Additional file 9. Neutral xylo-oligosaccharides released by CuGE and GH10 endo-xylanase after treatment of LRP. [file 13068_2018_1075_MOESM9_ESM.docx]

Additional file 9


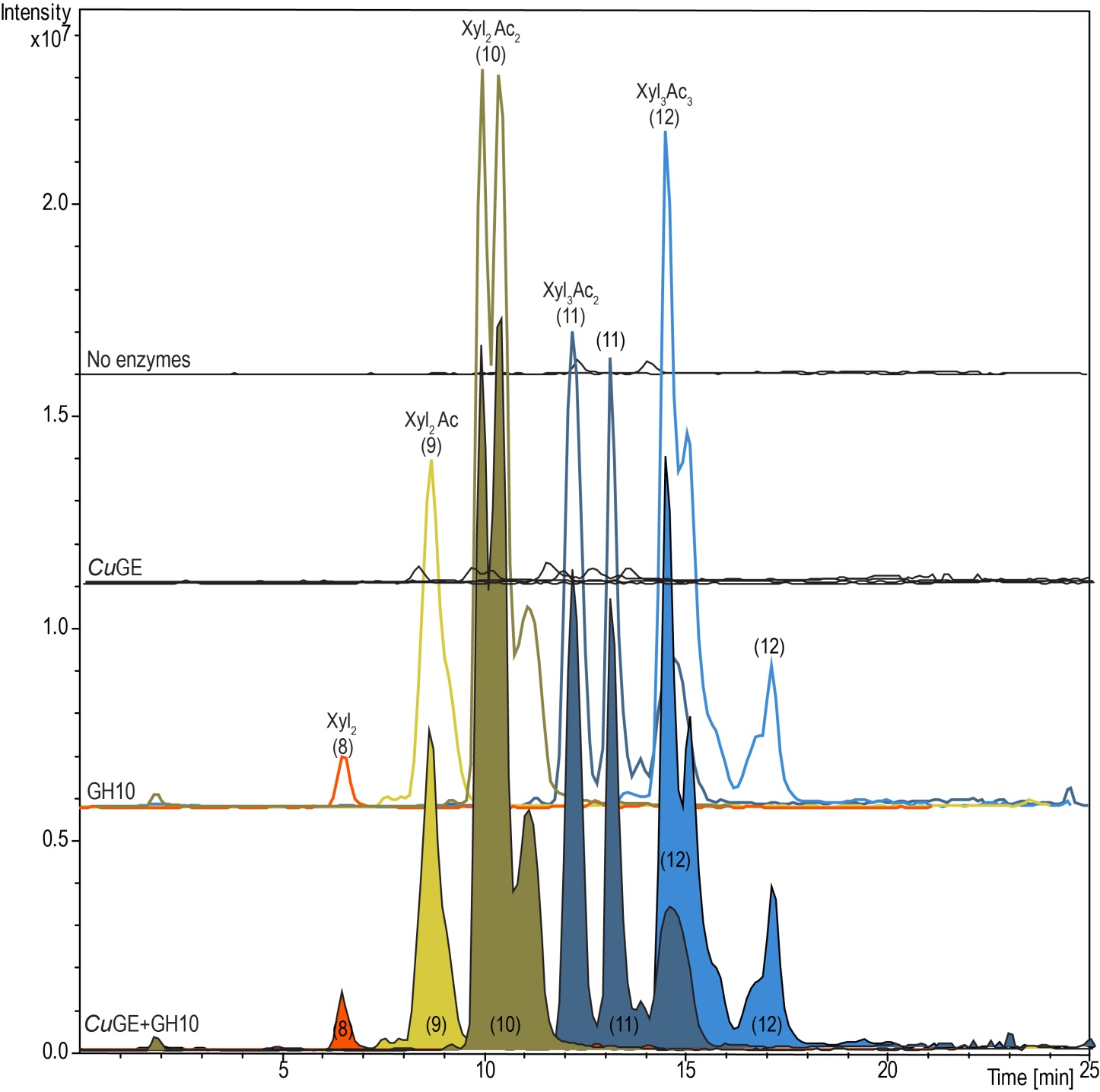


Release of neutral xylo-oliogsaccharides after treatment of LRP with *Cu*GE and GH10 alone and in combination analysed by LC-MS in MRM-mode. The product profiles show a mixture of acetylated xylo-oligos ranging from DP 2 to DP 3 each product assigned with a number and overall compound composition according to the molecular mass. Each compound mass gives rise to several peaks, indicating differences in substitution pattern.
