# Supplementary material for: The natural catalytic function of CuGE glucuronoyl esterase in hydrolysis of genuine lignin–carbohydrate complexes from birch
Source: Biotechnol Biofuels. 2018 Mar 19;11:71. doi: 10.1186/s13068-018-1075-2 (PMC5858132; doi:10.1186/s13068-018-1075-2)
Supplement: Supplementary file 10 — Additional file 10. Chromatogram of CuGE hydrolysed LRP after saponification. [file 13068_2018_1075_MOESM10_ESM.docx]

# Additional file 10

**
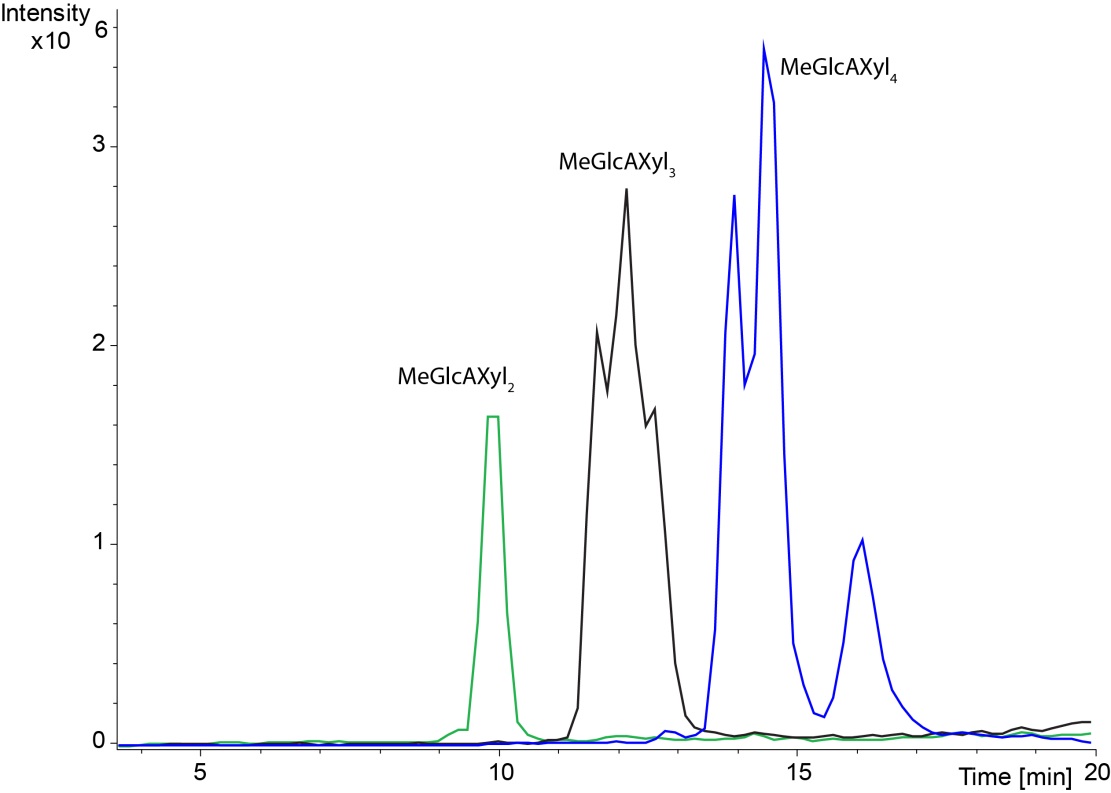
**

Chromatogram of *Cu*GE hydrolysed LRP. The enzyme hydrolysate was after enzyme treatment saponified with NaOH to remove all acetylations. The chromatogram shows that several peaks appear for each compound mass indicating structural isomers eluting at different retention times. The chromatograms suggest that *Cu*GE can release products with the 4-*O*-methyl-glucuronoyl substitution in various positions.
