# Supplementary material for: The natural catalytic function of CuGE glucuronoyl esterase in hydrolysis of genuine lignin–carbohydrate complexes from birch
Source: Biotechnol Biofuels. 2018 Mar 19;11:71. doi: 10.1186/s13068-018-1075-2 (PMC5858132; doi:10.1186/s13068-018-1075-2)
Supplement: Supplementary file 11 — Additional file 11. MS/MS of charged products released by by CuGE and GH10 endo-xylanase after treatment of LRP. [file 13068_2018_1075_MOESM11_ESM.docx]

**Additional file 11**


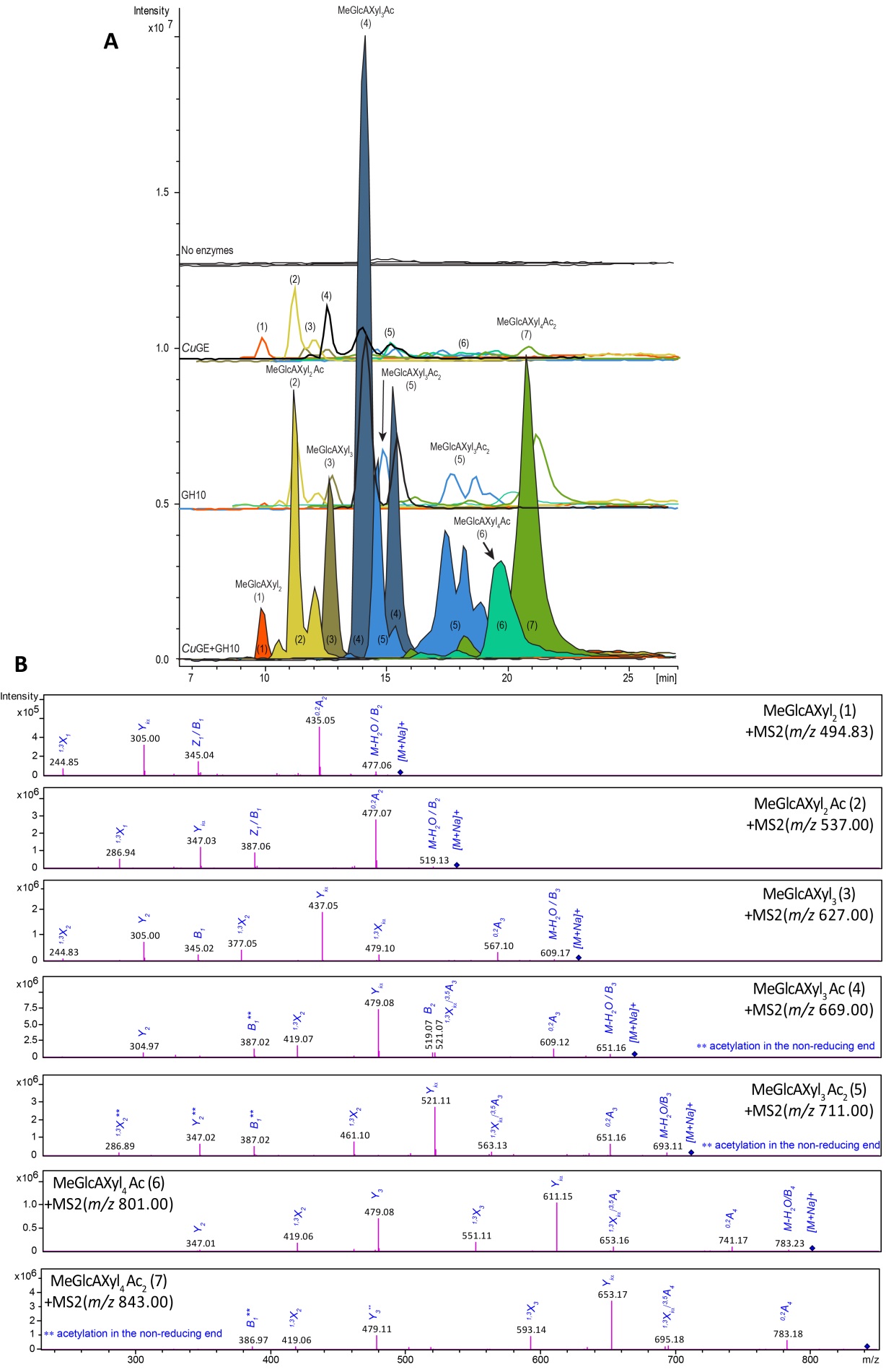


# MS/MS of charged products from incubation of GH10 endo-xylanase and *Cu*GE on LRP. Panel A shows the LC-MS chromatograms of charged products released by GH10 endo-xylanase and *Cu*GE incubated on LRP. Several peaks with the same *m/z*-value occur and these peaks are color coded and numbered accordingly. Products are named: (1): Aldotriuronic acid, MeGlcAXyl_2_ (2): Aldotriuronic acid with one acetylation, MeGlcAXyl_2_Ac (3): Aldotetrauronic acid, MeGlcAXyl_3_ (4): Aldotetrauronic acid with one acetylation, MeGlcAXyl_3_Ac (5): Aldotetrauronic acid with two acetylations, MeGlcAXyl_3_Ac_2_ (6): Aldopentauronic acid with one acetylation, MeGlcAXyl_4_Ac (7): Aldopentauronic acid with two acetylations, MeGlcAXyl_4_Ac_2_. Structural differences with respect to acetylation pattern and position are most likely the primary reasons why the same masses occur in several peaks. Panel B contains an MS/MS spectrum from each compound (1-7). The spectra are averaged across the entire elution period for each compound mass. The mother ion is represented by the sodium adduct [M+Na]^+^ and marked by a blue diamond. Fragment ions are also observed as sodium adducts and are named according to Domon & Costello nomenclature [1]. Due to isobaric masses certain fragment ions may have more than one possible origin and in such cases several possible fragments are listed and separated by (/) above the fragment peak. However, the naming of fragmentation ions is done under the general assumption that the GH10 endo-xylanase has a preference for generating aldouronic acid products with the glucuronoyl substitution in the non-reducing end. However, *Cu*GE may not necessarily possess the same preferences and hence some fragment ions may originate from products with the glucuronoyl substitution positioned elsewhere. The fragmentation spectra contain several glycosidic bond cleavages and this is related to varying positions of acetylations. Some glycosidic bond cleavages are dependent on specific positions of acetylations and these are marked by **. Also the α-linkage to the glucuronoyl substitution is prone for fragmentation (Y_iα_). The _i_ indicates that the glucuronoyl α-linkage might be positioned anywhere on the xylo-oligosaccharide and still result in the same fragment.

1. Domon B, Costello CE. A systematic nomenclature for carbohydrate fragmentations in FAB-MS/MS spectra of glycoconjugates. Glycoconj J. 1988;5:397–409.
