# Supplementary material for: The natural catalytic function of CuGE glucuronoyl esterase in hydrolysis of genuine lignin–carbohydrate complexes from birch
Source: Biotechnol Biofuels. 2018 Mar 19;11:71. doi: 10.1186/s13068-018-1075-2 (PMC5858132; doi:10.1186/s13068-018-1075-2)
Supplement: Supplementary file 12 — Additional file 12. MS/MS of neutral products released by by CuGE and GH10 endo-xylanase after treatment of LRP. [file 13068_2018_1075_MOESM12_ESM.docx]

# **Additional file 12**


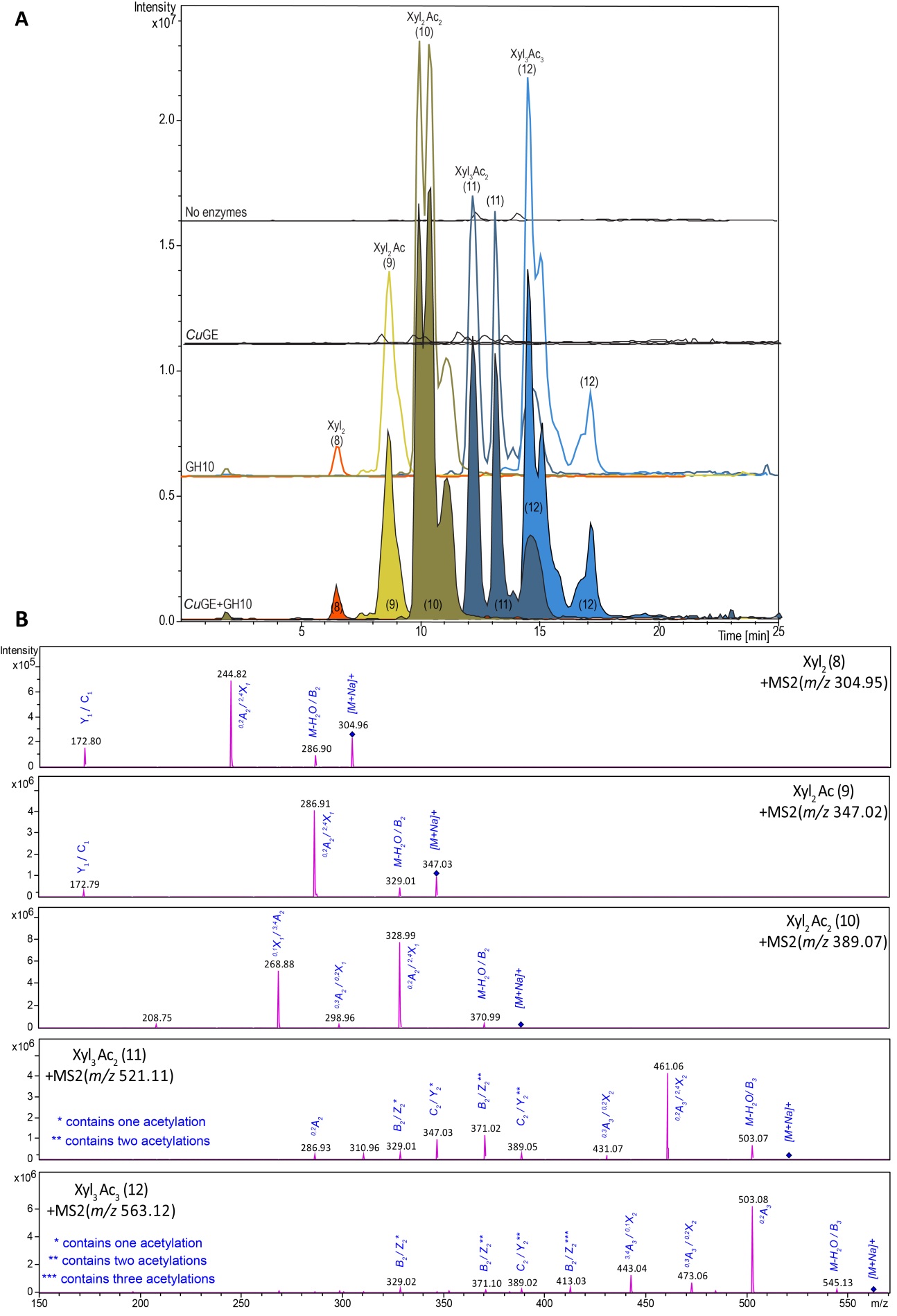


# MS/MS of neutral products from incubation of GH10 endo-xylanase and *Cu*GE on LRP. Panel A shows the LC-MS chromatograms of neutral products released by GH10 endo-xylanase and *Cu*GE incubated on LRP. Several peaks with the same *m/z*-value occur and these peaks are color coded and numbered accordingly. Since xylose is by far the most dominating monosaccharide in the overall composition of the substrate, the product peaks will also be dominated by xylooligosaccharides even though arabinosyls may also occur in minor amounts in the structures. Arabinosyls and xylosyls cannot be distinguished on the current level of analysis. Hence, the products are named as if they exclusively consist of linear xylosyl moieties with various degree of acetylation. (8): Xylobiose, Xyl_2_ (9): Xylobiose with one acetylation, Xyl_2_Ac (10): Xylobiose with two acetylations, Xyl_2_Ac_2_ (11): Xylotriose with two acetylations, Xyl_3_Ac_2_ (12): Xylotriose with three acetylations, Xyl_3_Ac_3_. Structural differences with respect to acetylation pattern and position are most likely the primary reasons why the same masses occur in several peaks. Panel B contains an MS/MS spectrum from each compound (8-12). The spectra are averaged across the entire elution period for each compound mass. The mother ion is represented by the sodium adduct [M+Na]^+^ and marked by a blue diamond. Fragment ions are also observed as sodium adducts and are named according to Domon & Costello nomenclature [1]. Due to isobaric masses (the compounds are identical in both the reducing and non-reducing end) several fragment ions may have more than one possible origin and in such cases several possible fragments are listed and separated by (/) above the fragment peak. The spectra are dominated by cross-ring fragments and to a lesser extent on glycosidic bond cleavages. Previous studies have shown that linear xylo-oligosaccharides primarily makes A-fragments and that these are indicative for β-(1→4)-linked backbone [2, 3]. Practically no loss of acetylations are observed as no loss of 102 Da is observed from ^0,2^A fragments [3]. The exact position of acetylations cannot be established at this level of analysis, and in cases where several of the same B or Z ions occur (e.g. compound 11 + 12, marked with *), the differences in mass is caused by varying position of the acetylations.

1. Domon B, Costello CE. A systematic nomenclature for carbohydrate fragmentations in FAB-MS/MS spectra of glycoconjugates. Glycoconj J. 1988;5:397–409.

2. Reis A, Coimbra MA, Domingues P, Ferrer-Correia AJ, Domingues MRM. Structural characterisation of underivatised olive pulp xylo-oligosaccharides by mass spectrometry using matrix-assisted laser desorption/ionisation and electrospray ionisation. Rapid Commun Mass Spectrom. 2002;16:2124–32.

3. Bauer S. Mass Spectrometry for Characterizing Plant Cell Wall Polysaccharides. Front Plant Sci. 2012;3:45.
