# Supplementary material for: The natural catalytic function of CuGE glucuronoyl esterase in hydrolysis of genuine lignin–carbohydrate complexes from birch
Source: Biotechnol Biofuels. 2018 Mar 19;11:71. doi: 10.1186/s13068-018-1075-2 (PMC5858132; doi:10.1186/s13068-018-1075-2)
Supplement: Supplementary file 13 — Additional file 13. Release of ferulic acid by CuGE from water insoluble wheat arabinoxylan. [file 13068_2018_1075_MOESM13_ESM.docx]

Additional file 13


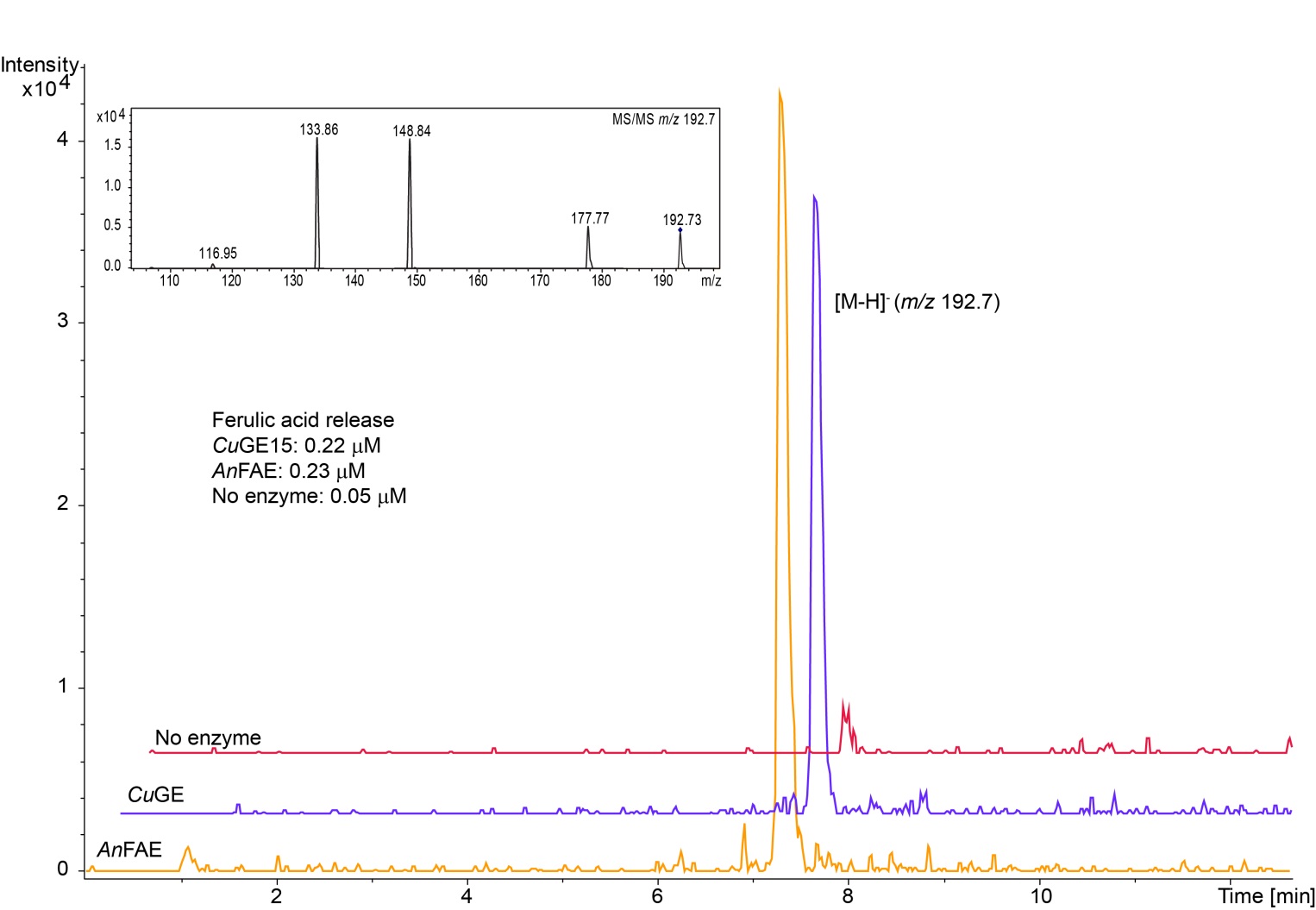


Release of ferulic acid by *Cu*GE from water insoluble wheat arabinoxylan detected by LC-MS after overnight incubation. Ferulic acid esterase from *Aspergillus nidulans (An*FAE) was included as positive control (see additional file 2 for more details). Fragmentation ions of the detected peak are shown in the top left box, resembling the expected fragmentation pattern for ferulic acid.
