# Supplementary material for: The natural catalytic function of CuGE glucuronoyl esterase in hydrolysis of genuine lignin–carbohydrate complexes from birch
Source: Biotechnol Biofuels. 2018 Mar 19;11:71. doi: 10.1186/s13068-018-1075-2 (PMC5858132; doi:10.1186/s13068-018-1075-2)
Supplement: Supplementary file 14 — Additional file 14. Complete list of MS precursors and MS/MS fragmentation ions for MRM. [file 13068_2018_1075_MOESM14_ESM.docx]

Additional file 14

Complete list of MS precursors and MS/MS fragmentation ions of enzyme reaction products in MRM-mode used for preparation of figure 1 and additional files 9-12.

| Analyte | Precursor *m/z* [M+Na]^+^ | MS/MS fragmentation ions *m/z* [M+Na]^+^ |
| --- | --- | --- |
| MeGlcAXyl_2_ | 495 | 245; 305; 327; 345; 403; 419; 435; 448; 458; 477 |
| MeGlcAXyl_3_ | 627 | 245; 286; 305; 345; 377; 437; 419; 479; 535; 567 |
| Reduced MeGlcAXyl_3_ | 629 | 287; 307; 345; 439; 481; 549; 567; 593; 606 |
| MeGlcAXyl_4_ | 759 | 287; 345; 305; 345; 377; 419; 437; 506; 509; 569; 609; 627; 699; 741 |
| Xyl_2_ | 305 | 173; 245; 287; 305 |
| Xyl_3_ | 437 | 287; 305; 347; 377; 419; 437 |
| Xyl_4_ | 569 | 245; 305; 377; 419; 437; 479; 509; 511 |
