# Supplementary material for: The natural catalytic function of CuGE glucuronoyl esterase in hydrolysis of genuine lignin–carbohydrate complexes from birch
Source: Biotechnol Biofuels. 2018 Mar 19;11:71. doi: 10.1186/s13068-018-1075-2 (PMC5858132; doi:10.1186/s13068-018-1075-2)
Supplement: Supplementary file 15 — Additional file 15. Complete list of adducts used for quantification of enzyme reaction products. [file 13068_2018_1075_MOESM15_ESM.docx]

Additional file 15

Complete list of adducts used for quantification of enzyme reaction products and their corresponding retention times. *the *m/z* 443 appears as a pseudo-ion derived from *m/z* 302 as it fragments to *m/z* 302 only.

| Analyte | *m/z* [M+Na]^+^ | *m/z* [M+NH_4_]^+^ | *m/z* [2M+Na+NH_4_]^2+^ | *m/z* [M+Na+NH_4_]^2+^ | *m/z* [M+H]^+^ | Retention time (min) |
| --- | --- | --- | --- | --- | --- | --- |
| MeGlcAXyl_2_ | 495 | 490 | 492 | - | - | 9.5-10 |
| MeGlcAXyl_3_ | 627 | 622 | 624 | - | - | 12-13 |
| Reduced MeGlcAXyl_3_ | 629 | 624 | - | - | 607 | 15.5-16.5 |
| MeGlcAXyl_4_ | 759 | 754 | - | 388 | - | 11.25-11.7 |
| Xyl_2_ | 305 | 300 | 302 (443*) | - | - | 6.25-7 |
| Xyl_3_ | 437 | 432 | 434 | - | - | 8.75-9.75 |
| Xyl_4_ | 569 | 564 | 566 | - | - | 10.9-11.7 |
